# Plasma levels of adhesion molecules are elevated in dermatomyositis-interstitial lung disease and associated with low paraoxonase-1 activity

**DOI:** 10.1186/s13075-025-03520-z

**Published:** 2025-03-08

**Authors:** Sangmee Sharon Bae, Ani Shahbazian, Jennifer Wang, Daniela Markovic, Tiffany De Leon, Yuna Lee, Srinivasa T. Reddy, Christina Charles-Schoeman

**Affiliations:** 1 UCLA David Geffen School of Medicine, Departmen of Medicine, Division of Rheumatology, University of California, 1000 Veteran Ave, Rm 32-59, Los Angeles, CA 90095 USA; 2 UCLA Department of Medicine Statistics Core, Los Angeles, USA; 3 UCLA David Geffen School of Medicine, Department of Medicine, Division of Cardiology, Los Angeles, USA

**Keywords:** Idiopathic inflammatory myopathy, Dermatomyositis, Polymyositis

## Abstract

**Objective:**

To evaluate circulating levels of intercellular cell adhesion molecule-1 (ICAM-1), vascular cell adhesion molecule-1 (VCAM-1) in patients with dermatomyositis (DM) and DM associated interstitial lung disease (DM-ILD).

**Methods:**

We performed a cross-sectional study in plasma samples from DM patients and matched healthy controls. Plasma ICAM-1 and VCAM-1 (CAM) levels were measured by ELISA. The activity of paraoxonase-1 (PON1), a high density lipoprotein (HDL) associated antioxidative enzyme was measured using paraoxonase, arylesterase and lactonase assays. Association analysis was performed between clinical predictors and CAM levels. We analyzed whether CAM levels have a mediating role in the association between PON1 activity and IIM outcomes using causal mediation analysis.

**Results:**

Plasma samples from 83 DM patients with anti-Jo1 (*n* = 24), MDA5 (*n* = 29), and TIF1gamma (*n* = 30) and 28 age and sex matched healthy controls were analyzed. Plasma CAM levels were significantly higher in DM patients compared to controls. CAM levels were particularly higher in anti-MDA5 + DM patients compared to other autoantibody groups and in DM-ILD compared to DM without ILD. Higher ICAM-1 levels correlated low PON1 lactonase activity as well as worse restrictive lung physiology in multivariate models. Mediation analysis showed that 54% of the effect of low lactonase on worse DLCO was mediated through ICAM-1.

**Conclusion:**

Plasma CAM levels were higher in DM patients compared to healthy controls, particularly in DM patients with ILD. Our analyses support a pathway of low PON1 lactonase activity representing poor HDL function with low protective capacity of microvessels allowing increased endothelial activation leading to DM and DM-ILD.

**Supplementary Information:**

The online version contains supplementary material available at 10.1186/s13075-025-03520-z.

## Key message

• Dermatomyositis (DM) patients had higher circulating ICAM-1 and VCAM-1 levels compared to healthy controls.

• Plasma CAM levels were particularly higher in anti-MDA5 + patients and in DM with interstitial lung disease (ILD).

• Our results support a potential pathway in which low PON1 associates with DM and DM-ILD partly mediated by increased endothelial activation.

**Supplementary Information:**

The online version contains supplementary material available at 10.1186/s13075-025-03520-z.

## Introduction

Idiopathic inflammatory myopathies (IIM) are a heterogenous group of autoimmune diseases characterized by chronic inflammation of primarily the skeletal muscle but also a wide range of extra-muscular manifestations. Interstitial lung disease (ILD) is reported in 5–65% in IIM cohorts and is a leading cause of morbidity and mortality [1, 2]. Damage to the vascular endothelium is implicated in the pathogenesis of IIM and its associated ILD (IIM-ILD), with microvascular involvement most described in the dermatomyositis (DM) subtype [3].

Cell adhesion molecules (CAM) are glycoproteins expressed on the surface of various cells and are responsible for the transmigration of leukocytes to the vascular intima promoting endothelial damage and inflammation [4, 5]. CAM levels serve as markers for endothelial activation and vascular inflammation in studies of atherosclerotic cardiovascular disease [6, 7]. In IIM, increased levels of intercellular cell adhesion molecule-1 (ICAM-1) and vascular cell adhesion molecule-1 (VCAM-1) have been described in the vasculature of DM and PM muscle [8, 9]. Circulating levels if ICAM-1 and VCAM-1 are also elevated in patients with adult IIM compared to healthy controls, although their association with disease severity or specific clinical features (skin, arthritis, ILD, cancer) vary between studies and remain unclear [10–13].

Paraoxonase-1 (PON1) is a high-density lipoprotein (HDL)-associated enzyme that promotes the antioxidant, anti-inflammatory function of HDL, and protects the vascular endothelium from damage due to oxidized phospholipids [14, 15]. We have previously demonstrated that impaired PON1 activity is associated with worse IIM disease activity and the presence of severe ILD [16]. However, whether the association between PON1 and IIM and IIM-ILD is mediated by vascular damage is unknown. We hypothesize that poor PON1 activity is associated with vascular damage evidenced by increased levels of circulating ICAM-1 and VCAM-1 leading to higher disease burden in IIM and IIM associated ILD, particularly in the DM subtype.

## Methods

### Study population

We performed a cross-sectional study using plasma samples collected from a single-center myositis cohort. We included DM patients with 3 myositis-specific autoantibodies (MSA) determined a priori: anti-Jo1 ab, anti-MDA5 ab and anti-TIF1 $\:\gamma\:\:$ ab, and age/sex matched healthy controls. All myositis patients met EULAR)/ACR Classification Criteria for adult IIM for at least “probable” IIM and subclass of DM [17]. All subjects gave written informed consent for the study approved by the Human Research Subject Protection Committee at UCLA.

### Clinical assessments

Clinical outcome measures were assessed at cohort enrollment. Laboratory studies including creatine phosphokinase (CPK) levels, inflammatory markers (high-sensitivity C-reactive protein [hsCRP], westergren erythrocyte sedimentation rate [ESR]), and fasting lipid profiles (total cholesterol, LDL-cholesterol [LDL-C], HDL-cholesterol [HDL-C], triglycerides) were performed by the UCLA clinical laboratory using standard methods.

The MSAs were analyzed using immunoprecipitation in 49 specimens (at the Oklahoma Medical Research Foundation) and 34 using other standardized immunoassays (line blot and ELISA).

Cardiovascular risk and health information including the presence of various myositis related clinical features, immunosuppressive medications were obtained by patient reported questionnaire and chart review. Myositis disease activity and damage were assessed using physician global scales by visual analog scale (VAS, 0–100 mm), 5-point Likert scale (0–4) and manual muscle testing of 8 muscle groups (MMT8, 0-150) [18].

ILD was defined by radiographic findings consistent with ILD on high-resolution chest CT (HRCT) per a radiologist read showing at least one of the following: (1) reticulation and fibrosis, (2) traction bronchiectasis, (3) honeycombing, or (4) ground glass opacification [19]. All HRCT and pulmonary function test (PFT) results closest to blood collection date were included in the analysis.

### Biomarker analysis

Plasma that was taken at cohort enrollment was stored − 80 °C until analyzed for biomarker assessments. Circulating levels of ICAM-1 and VCAM-1 were measured using enzyme linked immunosorbent assays (Invitrogen, ThermoFisher Scientific). Results were expressed in ng/mL.

PON1 activity was quantified using 3 different substrates (paraoxon, dihydrocoumarin, and phenylacetate) to assess its paraoxonase, lactonase, and arylesterase activities respectively as described previously [20].

### Statistical analysis

A student’s t-test or Wilcoxon rank-sum test was used to compare continuous variables, and a χ2 test or fisher’s exact test was used to compare categorical variables between DM patients and healthy controls. Biomarkers were compared between DM patients and healthy controls and between DM antibody subgroups using a Wilcoxon test.

Correlations were assessed between CAM levels and clinical/laboratory variables including PON1 enzyme activity using Spearman’s correlation for continuous variables and chi-square for categorical variables. Multivariate linear models were constructed to adjust for potential confounders. CAM levels were log transformed to fit linearity for all linear models.

Causal mediation analysis [21] was used to assess whether CAM levels have a mediating role in relationships between PON1 activity and IIM outcomes which have been observed in previous studies (schema for mediation analysis, Supplementary Fig. 1) [16]. We used the ‘mediate’ package in R to perform a series of regression models that estimate coefficients between the exposure and the outcome and compute whether inclusion of the mediator alters the effects of the exposure on the outcome. The analysis dissects the total effect of the exposure on the outcome to direct and indirect effects, indirect effect being the effect of the exposure on the outcome through the mediator while direct effect is the exposure on the outcome absent the mediator. We fitted each model with PON1 activity as the exposure and the following IIM outcomes: DM (vs. control), ILD yes (vs. no), FVC (% predicted), DLCO (% predicted). All models were adjusted for age. All continuous variables, including the PON1 markers (lactonase, arylesterase, paraoxonase) and mediators (ICAM-1, VCAM-1), were log transformed to fit linearity and standardized to have a mean of zero and effect size was calculated for every standard deviation (SD) increase of the exposure.

All reported *p*-values are based on two sided tests, and < 0.05 was considered statistically significant. Data was processed and analyzed using SAS version 9.4 (SAS institute, Cary, NC) and R (version 4.4.1).

## Results

### Clinical characteristics of DM patient group compared to healthy controls

A total of 83 patients with DM including patients with anti-Jo1 (*n* = 24), anti-MDA5 (*n* = 29), and anti-TIF1gamma (*n* = 30) were compared to 28 healthy controls (Table 1). DM patients had a mean age of 48 years, were 78% female, 64% White, 14% Hispanic, and 6% Black. Cardiovascular risk factors were similar in the two groups except for diabetes which was more common in the DM patients. Traditional lipid panels showed higher total cholesterol, LDL-C and triglycerides in DM patients compared to controls.

**Table 1:** Comparison between DM and controls

	DM (*n* = 83)	Control (*n* = 28)
Age, years	48 $\:\pm\:$ 15	44 $\:\pm\:$ 13
Sex, female	65(78)	21(75)
Ethnicity, Hispanic	12(14)	6(21)
Race, White	58(70)	19(68)
Black	5(6)	1(4)
Asian	20(24)	8(29)
VCAM-1, ng/mL	2931[2302–4376]*	**2158[1597–2526]**
ICAM-1, ng/mL	500[375–653]*	**359[305–452]**
Paraoxonase, U/ml	461[282–934]	463[269–810]
Arylesterase, U/ml	203[154–273]	240[32–304]
Lactonase, U/ml	16[11–22]*	**23[19–31]**
hsCRP, mg/L	1.5[0.6–5.3]	1.2[0.3–2.6]
ESR, mm/hr	27[13–49]*	**8[3–19]**
Total cholesterol, mg/dl	210[179–246]*	**183[161–205]**
LDL-C, mg/dl	117[92–158]*	**104[77–130]**
HDL-C, mg/dl	61[48–78]	57[52–68]
Triglyceride, mg/dl	151[94–214]*	**87[73–162]**
CVD risk factors		
History of MI	1(1)	1(4)
History of stroke	4(5)	0
Hypertension	16(19)	2(8)
Hyperlipidemia	10(12)	4(15)
Diabetes	8(10)*	**0**
FHx of premature MI	6(10)	6(23)
Ever smoker	12(18)	1(8)
BMI	26[23–30]	26[22–30]
Statin	9(11)	2(9)
**IIM characteristics**		
Disease duration, months	19[8–60]	
MSA group, Jo-1 ab	26	
MDA5 ab	29	
TIF1r ab	30	
ILD	44(53)	
Cancer	16(18)	
MD global activity VAS, 0-100	46 $\:\pm\:$ 21	
MD global activity likert, 0–4	2[1–2]	
MD global damage VAS, 0-100	31 $\:\pm\:$ 21	
MD global damage likert, 0–4	1[1–2]	
MMT8, 0-150	150[144–150]	
CPK, U/L	78[52–165]	
Aldolase, U/L	6.0[4.4–8.1]	

Values are in mean $\:\pm\:$ SD for non-skewed data, median[IQR] for skewed data

*p < 0.05 by student’s t test for non-skewed data and Wilcoxon test for skewed data, chi square test for categorical data

Abbreviations: CVD, cardiovascular disease; FHx of premature MI, family history of myocardial infarction in male first degree relative before age of 55 and female relative before age 65; MSA, myositis specific antibody; VAS visual analog scale

Median disease duration was 19 months, 53% of DM patients had ILD and 18% had a history of cancer. Patients had moderate disease activity and damage according to MD global disease activity and damage scores (MD global activity VAS 46 $\:\pm\:$ 21, MD global damage VAS 31 $\:\pm\:$ 21, mean $\:\pm\:$ SD).

### ICAM-1 and VCAM-1 levels were higher in DM patients, especially anti-MDA5 + DM patients

When analyzed as a group, irrespective of MSA subtype, DM patients had significantly higher plasma ICAM-1 and VCAM-1 levels compared to healthy controls (Table 1). Bivariate analysis showed inflammatory markers, triglyceride and diabetes were also positively correlated with ICAM-1 and VCAM-1 levels (Table 2). Multivariate linear models with ICAM-1 and VCAM-1 as outcome variables were constructed in which each model was adjusted for variables that were significantly different between DM and controls. The DM diagnosis (vs. controls) remained significantly associated with higher ICAM-1 and VCAM-1 levels in both multivariate models (Supplementary Table 1).

**Table 2:** Associations between ICAM/VCAM with clinical/laboratory variables in DM and controls (*n* = 111)

Continuous variables		ICAM-1		VCAM-1	
		$\:\varvec{\rho\:}$	*P* value	$\:\varvec{\rho\:}$	*P* value
Age		0.14	0.16	0.10	0.29
BMI		0.07	0.49	0.02	0.86
Paraoxonase, U/ml		0.10	0.29	-0.13	0.19
Arylesterase, U/ml		-0.07	0.46	0.02	0.87
Lactonase, U/ml		**-0.21**	**0.03**	**-0.21**	**0.03**
hsCRP, mg/L		**0.23**	**0.01**	0.14	0.15
ESR, mm/hr		**0.28**	**0.003**	**0.33**	**0.001**
Total cholesterol, mg/dl		0.07	0.49	0.03	0.73
LDL-C, mg/dl		-0.01	0.89	0.02	0.81
HDL-C, mg/dl		-0.18	0.07	-0.16	0.10
Triglyceride, mg/dl		**0.34**	**0.0004**	**0.23**	**0.02**
		**ICAM-1**		**VCAM levels-1**	
**Categorical**	**N**	**Variable Yes** Median[IQR]	**Variable No** Median[IQR]	**Variable Yes** Median[IQR]	**Variable No** Median[IQR]
Sex, female	86	427[352–576]	478[342–665]	2648[2077–3473]	2935[1886–5559]
Ethnicity, Hispanic	17	411[337–720]	451[350–583]	2652[2117–3766]	2726[2050–3768]
Race, White	69	414[346–572]	509[347–657]	2747[2069–3768]	2596[1931–3542]
Black	6	509[431–623]	431[346–608]	1943[1066–3054]^	2737[2108–3798]
Asian	19	512[335–675]	426[346–576]	2641[2155–4273]	2737[2047–3745]
CVD risk factors					
History of MI	2	490[476–503]	446[346–628]	2802[1228–4375]	2737[2086–3751]
History of CVA	4	466[390–733]	447[346–609]	3551[2364–10412]	2737[2049–3748]
Hypertension	18	458[382–589]	446[342–631]	2723[1999–4212]	2737[2085–3751]
Hyperlipidemia	14	416[297–501]	451[354–630]	2457[1713–3981]	2737[2154–3785]
Diabetes	8	549[508–1017]*	**429[344–583]**	3892[3005–5595]*	**2679[1996–3727]**
FHx of premature MI	12	377[305–537]	457[376–630]	2809[1895–3805]	2737[2060–3740]
Ever smoker	14	465[370–636]	457[357–615]	2380[2170–3410]	2903[2102–3842]
Statin	11	451[383–703]	446[342–589]	2602[1771–4413]	2747[2052–3784]

For continuous variables, reported Spearman’s correlation coefficients, for categorical variables reported median [IQR] ICAM/VCAM levels of subjects applicable to the row category vs. subjects not applicable to the category compared using Wilcoxon rank-sum test

*p < 0.05, ^ is *p* < 0.1

**Fig. 1:**
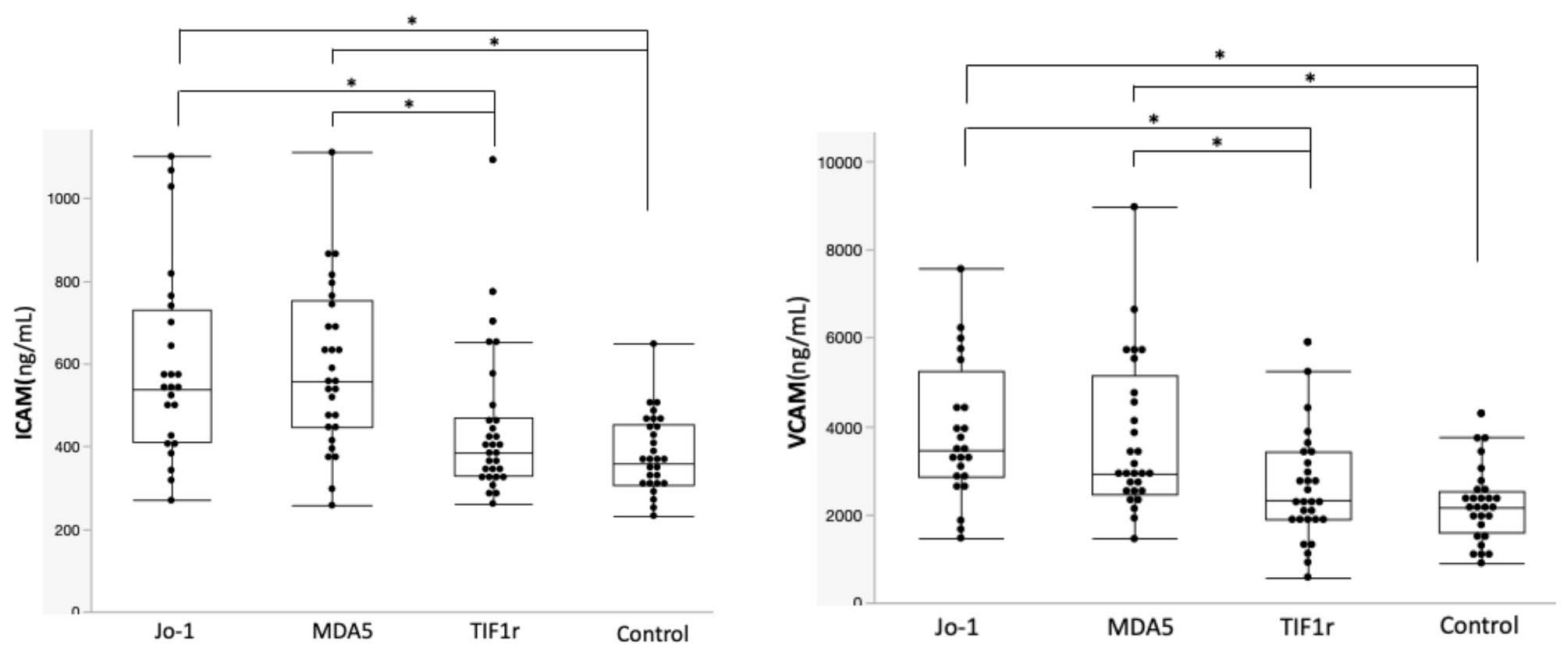
Plasma ICAM-1 and VCAM-1 levels in DM patients and Controls (*n* = 111) *p < 0.01 by Wilcoxon test Anti-Jo1 (*N* = 24), anti-MDA5 (*N* = 29), anti-TIF1 (*N* = 30) and Age/sex matched healthy controls (*N* = 28)

DM patients were further divided into MSA subgroups which showed that patients with anti-Jo1 and anti-MDA5 antibodies had significantly higher ICAM-1 and VCAM-1 levels compared to both healthy controls and anti-TIF1gamma positive DM patients (Fig. 1). In multivariate models, both ICAM-1 and VCAM-1 levels were significantly higher in anti-MDA5 ab + DM patients compared to other DM patients and controls while the anti-Jo1 + DM comparison was no longer statistically significant (Supplementary Table 2). Triglycerides remained positively associated with higher ICAM-1 and VCAM-1 in all multivariate models (Supplementary Tables 1,2).

### ICAM-1 and VCAM-1 levels are higher in DM patients with ILD compared to DM patients without ILD

We explored the association of CAM levels with clinical features of myositis and myositis disease outcome measures within the DM cohort (*n* = 83, Table 3). In univariate analysis, ICAM-1 levels were higher in patients with ILD and in patients who had dyspnea or cough, and ICAM-1 levels correlated with worse FVC and DLCO Hg % predicted values. Higher ICAM-1 levels also significantly associated with shorter disease duration, higher aldolase and higher MD global damage scores but not with MD global activity scores (Table 3). Patients with a history of respiratory failure (ever requiring supplemental O2 use) had numerically higher ICAM-1 levels which did not reach statistical significance (*p* = 0.09).

**Table 3:** Associations between CAM levels and clinical/laboratory characteristics of only DM patients (*n* = 83)

Continuous		ICAM-1		VCAM-1	
		Correlation coefficient $\:\:\varvec{\rho\:}$	*P* value	Correlation coefficient $\:\:\varvec{\rho\:}$	*P* value
Disease duration, months		-0.24*	**0.03**	0.001	0.99
CPK, U/ml		-0.01	0.91	-0.01	0.92
Aldolase, U/ml		0.23*	**0.049**	0.15	0.18
MD activity VAS, 0-100		0.15	0.16	0.07	0.51
MD activity likert, 0–4		0.18	0.10	0.05	0.63
MD damage VAS, 0-100		0.35*	**0.001**	0.18	0.10
MD damage likert, 0–4		0.31*	**0.004**	0.05	0.63
MMT8, 0-150		-0.07	0.62	-0.10	0.48
FVC, % predicted		-0.42*	**0.0008**	-0.21	0.12
DLCO Hg, % predicted		-0.40*	**0.004**	-0.11	0.41
Prednisone dose, mg/day		0.17	0.16	0.25*	**0.03**
		**ICAM-1**		**VCAM-1**	
**Categorical**	**N**	**Variable Yes** Median[IQR]	**Variable No** Median[IQR]	**Variable Yes** Median[IQR]	**Variable No** Median[IQR]
*Comorbidities*					
ILD	42	577[492–764]*	**395[336–509]**	3350[2730–4939]*	**2557[1923–3687]**
Cancer	15	503[342–703]	498[385–638]	3273[2324–4375]	2095[2258–4340]
Pulmonary hypertension	5	577[423–730]	498[374–652]	2929[2047–4269]	2933[2288–4385]
Coronary artery disease/ atherosclerosis	6	475[382–831]	499[372–653]	4997[2676–5983]	2929[2237–3862]
*Myositis manifestations*, ever					
Calcinosis	8	490[422–747]	499[370–652]	3279[2331–4510]	2879[2303–4121]
Hoarseness	17	530[335–661]	484[377–661]	2935[2455–3996]	2904[2161–4425]
Dysphagia	28	553[421–702]	447[373–641]	3464[2317–5040]	2868[2243–3853]
Proximal muscle weakness	69	503[386–689]	455[332–634]	3089[2308–4437]	2574[2225–3315]
Neuropathy	17	548[416–662]	466[373–661]	3233[2577–4915]	2861[2225–4385]
Periungal erythema	20	426[330–626]	519[390–700]	3039[2207–3971]	2931[2302–4413]
Skin ulcerations	13	459[382–683]	521[374–661]	3438[2203–4993]	2873[2288–3998]
Arthritis	54	521[395–696]	420[331–646]	3119[2419–4481]	2829[2092–3862]
Raynauds	16	449[361–699]	503[375–652]	3542[2969–4510]^	2829[2172–4121]
Mechanic’s hands	16	540[434–626]	459[370–684]	3253[1976–4298]	2878[2302–4375]
Fever	12	608[487–789]^	459[370–652]	4012[3192–5657]*	**2829[2171–3853]**
Oral ulcers	8	567[414–845]	496[370–652]	3093[1578–4595]	2931[2314–4375]
Alopecia	18	558[373–696]	496[376–652]	3155[1875–5720]	2931[2335–3863]
Cough	25	628[475–780]*	**427[364–580]**	3318[2324–4993]	2873[2215–3934]
Dyspnea	55	531[406–743]*	**386[330–543]**	3319[2602–4753]*	**2417[1883–2417]**
Respiratory Failure (supplemental O2 use)	13	643[454–840]^	465[372–633]	3318[2612–4080]	2861-2225-4446]
Fatigue	33	472[382–608]	513[372–697]	2748[1982–5231]	3010[2448–3998]
Weight loss	18	558[476–710]^	447[368–653]	3687[2614–5550]^	2854[2207–3798]
Myalgias	23	529[405–700]	497[359–650]	3745[2052–5500]	2861[2305–3729]
Dry eyes	9	518[414–599]	497[369–687]	4121[2455–5922]	2903[2161–3857]
Dry mouth	12	509[415–565]	496[370–695]	3751[2332–5686]	2929[2243–3853]
*Medications*, at time of blood draw					
Intravenous or subcutaneous immunoglobulin	37	472[386–673]	526[364–661]	3395[2455–4993]^	2824[2051–3822]
Mycophenolate Mofetil	36	524[397–684]	472[367–653]	2933[2515–4059]	2879[1929–4413]
Rituximab	18	524[406–668]	496[371–668]	3472[2402–5682]	2929[2207–3833]
Cyclophosphamide	6	659[449–788]	496[371–647]	3611[2482–5121]	2929[2273–4248]
Azathioprine	4	527[365–933]	497[379–653]	3314[2967–5340]	2892[2189–4312]
Methotrexate	14	401[319–519]*	**529[386–697]**	2188[1669–3531]*	**3089[2417–4606]**
Hydroxychloroquine	16	466[362–701]	500[375–643]	2989[2263–3734]	2935[2302–4460]
Prednisone	62	511[391–661]	413[336–713]	3012[2340–3934]	2679[2030–5618]

Continuous variables, reported Spearman’s correlation coefficients ($\:\varvec{\rho\:}$) and *p* value marked as *for *p* < 0.05 or ^ for *p* < 0.10

Categorical variables reported median [IQR]. Comparison of CAM levels of subjects applicable to the row category vs. subjects not applicable to the category using Wilcoxon rank-sum test is marked as either *p < 0.05 or ^ *p* < 0.10

Higher VCAM-1 levels also associated with the presence of ILD and dyspnea (Table 3). Higher VCAM-1 was also associated with fever as a feature of their DM. Patients with Raynauds and history of weight loss as a DM feature had higher VCAM-1 levels, but did not meet statistical significance (*p* = 0.08 for both).

We compared clinical, laboratory variables and medications of DM patients with ILD (*n* = 42) to DM patients without ILD (*n* = 41, Supplementary Table 3). DM patients with ILD were significantly older, more likely to be male, have diabetes, and had higher hsCRP and triglycerides. Among medications, ILD patients had more use of mycophenolate, rituximab, cyclophosphamide, prednisone, and significantly less use of methotrexate.

Multivariate logistic regression models were used to further evaluate the association between CAM levels and the presence of ILD. Models were adjusted for predictors that were different between the ILD vs. no ILD group including medications (Supplementary Table 3). ILD remained significantly associated with higher ICAM-1 levels but not with VCAM-1 in multivariate models (Table 4). ILD also remained significantly associated with older age, male gender, higher triglyceride, mycophenolate and absence of methotrexate (Table 4).

**Table 4:** Association with ILD (vs. No ILD) using multivariate logistic regression models in DM only cohort (*n* = 83)

	Models with ICAM-1							Models with VCAM-1					
	OR	*P* value	OR	*P* value	OR	*P* value	OR		*P* value	OR	*P* value	OR	*P* value
ICAM-1	**13.8**	**< 0.01**	**6.72**	**0.02**	**9.63**	**< 0.01**	-		-	-	-	-	-
VCAM-1	-	-	-	-	-	-	2.15		0.12	1.54	0.45	1.07	0.87
Age	**1.04**	**< 0.01**	**1.04**	**0.01**	**1.05**	**< 0.01**	**1.04**		**0.01**	**1.05**	**0.01**	**1.05**	**0.01**
Sex, Female	**0.17**	**< 0.01**	**0.13**	**0.01**	**0.08**	**< 0.01**	**0.21**		**0.02**	**0.13**	**0.01**	**0.08**	**< 0.01**
Triglyceride	-	-	**1.01**	**0.01**	1.01	0.08	-		-	**1.01**	**0.005**	**1.01**	**0.02**
hsCRP	-	-	1.03	0.37	1.04	0.25	-		-	1.05	0.17	1.05	0.11
MMF	-	-	-	-	**5.97**	**< 0.01**	-		-	-	-	**4.65**	**0.01**

ILD (*n* = 42) is the outcome of all multivariate logistic models. Reported odds ratios per unit change. ICAM and VCAM were log transformed

Models adjusted for predictors associated with presence of ILD (vs. DM without ILD). MSA group was not included as it was highly correlated with the outcome variable. Diabetes produced unstable estimate (*n* = 0 in DM without ILD group) and was not included in multivariate models

Models are adjusted sequentially, first for age/sex, then age/sex/labs, then age/sex/labs/medications

In models including medications, one medication was included at a time (MMF, RTX, CYC, MTX, prednisone). MMF model included in table as a representative medication variable. ICAM-1 remained associated and VCAM-1 remained NOT associated with ILD outcome in models which included one of each following medication per model: RTX, CYC, MTX, prednisone. MTX has significant negative association with ILD (OR = 0.05, *p* = 0.001 in VCAM model, OR = 0.06, *p* = 0.003 in ICAM model), RTX, CYC, prednisone are NS

### Higher CAM levels are correlated with lower PON1 activity

Among functional assays of the PON1 enzyme, lactonase activity was significantly lower in DM patients compared to controls (Table 1). Arylesterase and paraoxonase activity were also numerically lower in DM patients but the difference did not reach statistical significance.

Lower lactonase activity of PON1 was significantly correlated with higher ICAM-1 and VCAM-1 levels (spearman’s $\:\rho\:=$ -0.21, *p* = 0.03 for both, Supplementary Fig. 2). PON1 activity by paraoxonase and arylesterase were not significantly correlated with CAM levels.

We performed multivariate linear regression in the DM cohort with lactonase as a predictor and CAM levels as the outcome adjusting for variables associated with CAM levels in univariate analysis. Higher ICAM-1 but not VCAM-1 levels remained associated with lower lactonase activity after multivariate adjustment (Supplementary Table 5).

### Mediation effects of CAM in the association between PON1 and DM diagnosis

In order to test our hypothesis that the relationship between poor PON1 activity and DM diagnosis is at least partially mediated by increased vasculopathy (identified by increased CAM levels), we performed causal mediation analysis (Table 5, Supplementary Fig. 1 for schema). The odds of being a DM patient (vs. control, ‘Outcome’) decreased by 15% (‘Total effect’ OR = 0.85) for every standard deviation (SD) increase of lactonase (‘Exposure’). Of this total decrease, 4% of the reduction in the odds of DM diagnosis (‘Indirect effect’ OR = 0.96) was mediated through ICAM-1 (‘Mediator’) and 11% decrease (‘Direct effect’ OR = 0.89) was *not* mediated through ICAM-1. The effect of lactonase that was mediated through ICAM-1 was 27% of the total effect and was statistically significant (*p* = 0.014). Similar mediation effects were also seen with VCAM-1. Odds of DM diagnosis decreased by 14% (total OR = 0.86) for each SD increase of lactonase, of which 14% of the total effect was mediated through VCAM-1 (*p* = 0.046). PON1 activity by arylesterase and paraoxonase assays did not have significant associations with DM diagnosis by logistic regression in the current analysis and no significant mediation effects were seen.

**Table 5:** Mediation effects of CAM

Exposure	Mediator	Outcome	Effect	OR[95% CI]	*P* value	% of total effect
Lactonase	ICAM-1	DM (vs. Control)	Indirect	0.96[0.92–0.99]	**0.014**	**27%**
			Direct	0.89[0.81–0.97]	**< 0.01**	73%
			Total	0.85[0.76–0.93]	**< 0.01**	
Lactonase	VCAM-1	DM (vs. Control)	Indirect	0.98[0.94–0.99]	**0.046**	**14%**
			Direct	0.88[0.80–0.97]	**< 0.01**	86%
			Total	0.86[0.78–0.95]	**< 0.01**	
		**Outcome** **(within DM only)**		$\:\beta\:$ **[95% CI]**		
Lactonase	ICAM-1	FVC	Indirect	1.92[-0.27 -4.97]	0.09	22%
			Direct	6.92[0.71–12.75]	**0.03**	78%
			Total	8.84[2.54–14.52]	**< 0.01**	
Lactonase	VCAM-1	FVC	Indirect	0.18[-0.94-1.75]	0.76	2%
			Direct	8.82[2.51–14.79]	**< 0.01**	98%
			Total	8.99[2.86–15.14]	**< 0.01**	
Lactonase	ICAM-1	DLCO	Indirect	2.47[0.15–5.94]	**0.03**	54%
			Direct	1.90[-3.93-7.77]	0.51	46%
			Total	4.37[-1.18-10.18]	0.13	
Lactonase	VCAM-1	DLCO	Indirect	0.12[-0.97-1.36]	0.86	3%
			Direct	4.34[-1.55-10.11]	0.14	97%
			Total	4.45[-1.66-10.23]	0.12	

All models adjusted for age. Paraoxonase, arylesterase, VCAM-1, ICAM-1 were log transformed to fit linearity

Effect size is for every standard deviation(SD) increase of the exposure. Indirect effect is the effect of the exposure on the outcome through the mediator, and the direct effect is the effect of the exposure on the outcome absent the mediator. Estimated coefficients and *p* values are from logistic regression with binary outcome (DM versus Control) reported with odds ratio (OR), and linear regression for continuous outcome (FVC, DLCO) with regression coefficient reported

### ICAM-1 level associates with DM-ILD severity

Among DM patients, those with ILD had lower CAM. To evaluate the relationship between CAM levels and ILD severity, multivariate linear models were constructed with FVC and DLCO within DM patients (Supplementary Table 4). Higher ICAM-1 was a significant predictor of lower FVC and lower DLCO in multivariate models, while VCAM-1 was not a significant predictor of these pulmonary outcomes.

Previous work has demonstrated the association between low PON1 and severe ILD [16]. Mediation analysis was performed with ILD severity as FVC and DLCO as an outcome and CAM as a mediator (Table 5). Increase in PON1 lactonase activity by 1 SD was associated with a 4.4 point increase in DLCO % predicted value (total effect). Among the association between lactonase and DLCO, 54% was mediated through ICAM-1 (indirect effect, *p* = 0.03) and 46% was not mediated through ICAM-1 (direct effect, *p* = 0.51). Increase in lactonase was significantly associated with higher FVC, and had a trend towards partial mediation through ICAM-1 (indirect effect 22%, *p* = 0.09), but most of the effect was not mediated through CAM.

## Discussion

In the current study, plasma ICAM-1 and VCAM-1 levels were higher in DM patients compared to matched healthy controls, and among DM patients CAM levels were higher in anti-MDA5 ab + patients and in patients with ILD.

Historically, studies have described increased CAM expression in endothelial cells of the microvessels in muscle tissue in IIM patients [9, 22, 23]. *Figarella* and colleagues reported muscle biopsies from IIM samples had marked increase in ICAM-1 expression, and each IIM subtype had distinct adhesion molecule profiles. DM muscle samples had ICAM-1 upregulation especially in vessels located at perimysial and perifascicular sites even in cases lacking inflammatory cells on the muscle biopsy [11].

Elevated soluble CAM levels have also been reported in the circulation of IIM patients by other groups and may suggest microvascular damage not only in the muscle but also as part of extra-muscular manifestations of the disease [10, 12, 13]. *Figarella* and colleagues investigated plasma levels of the adhesion molecules, P-selectin and E-selectin and showed higher circulating levels of these markers compared to controls in addition to abnormal expression of ICAM-1 in endothelial cells of DM muscle tissue [11].

Among DM patients in our cohort, plasma CAM levels were significantly higher in patients with ILD, and higher ICAM-1 correlated with more severe restrictive lung physiology. The importance of vasculopathy of the lung microvessels has been studied in other autoimmune disease-related ILDs. In patients with RA and SSc, serum E-selectin, ICAM-1 and endothelin-1 (ET-1) were increased in patients with ILD compared to patients without ILD and elevated levels correlated with worse FVC and DLCO [24]. Vasculopathy may play a role even in the early stages of developing lung fibrosis. A large study from community-dwelling adults showed circulating ICAM-1, VCAM-1 and P-selectin were independently associated with CT features and spirometric measures of subclinical ILD, increased rate of ILD hospitalization and ILD related death [25]. In IIM, cohort studies have demonstrated higher levels of circulating VCAM-1, ET-1, thrombomodulin, and plasminogen activator inhibitor-1 in IIM patients with ILD compared to IIM without lung disease [26, 27].

The majority of patients with anti-Jo1 or anti-MDA5 ab in the current work had ILD (88% of anti-Jo1, 72% in anti-MDA5), and these two antibody subgroups showed higher CAM levels compared to anti-TIF1 $\:\gamma\:$+ patients or controls. Myositis autoantibodies are often specific for myositis and rarely found in other diseases and studies have postulated that certain MSAs may have a pathogenic role in IIM. For example, stimulation of MDA-5, the antigen for anti-MDA5 ab, was shown to have profound effects on the vascular endothelium in human arterial endothelial cells and promote atherosclerosis in murine models [28]. Another in vitro study of sera from ani-Jo1+ patients with ILD induced ICAM-1 expression in cultured human microvascular endothelial cells derived from lung tissue which was significantly stronger than the response induced with sera from healthy controls or IIM patients with anti-SSA or anti-U1 RNP ab [29]. In our current study, anti-MDA5+ but not anti-Jo1+ remained significantly associated with higher CAM levels after multivariate adjustment. A Chinese DM cohort also found a significant difference in VCAM-1 levels between anti-MDA5 positive vs. negative patients but such difference by the presence or absence of the antibody was not seen in other antibody groups including antisynthetase ab, anti-TIF1 ab, anti-mi2 ab, and anti-NXP2 ab [26]. Further work is needed to elucidate whether autoantibodies are indeed pathogenic and whether endothelial activation and vasculopathy are caused by the autoantibodies themselves or other serum factors associated with autoantibodies.

HDL and its major antioxidant associated enzyme, PON1, play important roles in protection of the vascular endothelium [14, 15]. In the current work, low lactonase activity of the PON1 enzyme was significantly correlated with higher CAM levels, particularly ICAM-1. Such findings support our hypothesis that poor antioxidant function of HDL with low PON1 activity is associated with increased vascular damage. Previous work from our group described lower PON1 activity in DM patients compared to controls [16]. Here we performed mediation analysis to determine how much of the association between low PON1 activity and DM diagnosis was mediated through increased vascular damage and found a quarter of the effect of PON1 lactonase activity on DM was through ICAM-1.

The mediation effects of ICAM-1 were more pronounced in DM patients with ILD. Higher ICAM-1 levels were associated with lower FVC and DLCO in multivariate models in the DM cohort. While our current sample size was not sufficient to demonstrate significant differences in lactonase levels between ILD and no ILD patients, low PON1 lactonase activity associated with severe IIM-ILD (DLCO $\:\le\:$ 40%) compared to IIM patients with mild ILD or no ILD in a prior study with larger numbers [16]. Mediation analysis in our current work showed a significant portion of the association between lactonase and DLCO was mediated through ICAM-1, and a similar trend with FVC.

The protective role of the HDL particle in lung disease has been suggested in a few studies from large community-based cohorts. Associations between plasma lipoproteins with subclinical ILD in the Multi-Ethnic Study of Atherosclerosis cohort showed greater HDL-cholesterol and ApoA-1 (the major protein component of HDL) levels associated with less subclinical lung inflammation on CT and biomarkers of lung injury (SP-A and MMP-7) independent of demographics, smoking and inflammatory markers [30]. A retrospective analysis of the Atherosclerosis Risk in Communities cohort demonstrated low HDL cholesterol and higher triglyceride levels were strongly associated with increased risk of pneumonia hospitalizations [31]. While causality of the effects of HDL function on vasculopathy and ILD severity remains to be confirmed with mechanistic studies, our findings postulate a possible pathway in which poor HDL function with low protective capacity of microvessels in target organs such as muscle and lung, allows increased endothelial activation and vascular damage, that may perpetuate the development of DM and DM-ILD.

In addition to myositis autoantibodies, multivariate models in the current work revealed additional variables associated with ILD including older age, male sex and higher triglycerides. A retrospective study from an Italian cohort found that dyspnea at myositis onset and anti-Ro52 antibodies were predictive of the development of ILD, while demographics including age and sex were not [32]. In patients with hypertriglyceridemia or acute inflammatory states, lipoprotein composition is modified to have higher triglyceride content forming triglyceride-rich lipoproteins, and epidemiological and genetic studies support that such triglyceride-rich lipoproteins are an additional cause of cardiovascular mortality [33]. Circulating triglyceride-rich lipoproteins enhance endothelial inflammation and promote ICAM-1 and VCAM-1 expression [34, 35], and if occurring in microvessels in the lungs, this could be a potential mechanistic association between triglycerides and ILD. In addition, triglyceride-enriched HDL particles appear to be aberrant in their function including their protective anti-oxidative function [36].

There are limitations to our study. Our analysis was limited to DM patients with selected myositis autoantibodies from a single tertiary academic center. While our inclusion of pre-defined disease subgroups allowed us to investigate our hypothesis in relation to certain clinical phenotypes such as ILD, our findings may not be generalizable to other autoantibody groups. Also, as most patients with anti-Jo1 and anti-MDA5 ab had ILD, we were unable to differentiate the effects of autoantibodies from the effects of ILD. Future study with larger numbers including antibody positive patients both with and without ILD as well as other myositis autoantibody groups is needed. Multi-center studies with larger numbers may elucidate associations with additional clinical phenotypes and/or important clinical endpoints such as respiratory failure or mortality. Second, we analyzed associations from a single timepoint cross-sectionally. Additional work including longitudinal samples analyzed with repeated measures as an effort to elucidate the association between PON1 and CAM over time are underway. We used causal mediation analysis to draw a possible underlying pathway, however we acknowledge that experimental mechanistic work is needed to establish true causality. Third, most patients were on immunosuppressive therapy at the time of their biomarker assessment. Although we adjusted for medications as a potential confounder in our multivariate models, we are unable to account for the effect that treatment may have on biomarker levels. Future studies may evaluate pre-therapeutic as well as post-therapeutic levels. Lastly, in addition to CAM there are various molecular markers that represent endothelial injury at different junctures of the pathway. Cells or microparticles detached from the endothelium, selectin proteins induced by cytokine stimulation that is more specific to endothelial cells, coagulation factors including von Willebrand Factor which transports factor VIII in response to activation of the endothelium, cytokines and interferon related chemokines are additional biomarkers of vasculopathy that have been studied [37].

## Conclusions

We describe increased plasma ICAM-1 and VCAM-1 levels in DM patients compared to healthy controls, in particular in anti-MDA5 ab + DM and patients with ILD. Our results support a possible pathway that perpetuates vascular damage in DM and DM-ILD, that involves low PON1 enzyme lactonase activity representing low protective capacity of HDL for microvessels allowing increased endothelial activation and vascular damage. Longitudinal prospective studies of may be warranted to further evaluate the role of PON1 and vascular damage in the development and propagation of IIM and IIM-ILD.

## Electronic supplementary material

Below is the link to the electronic supplementary material.

Supplementary Material 1

## Data Availability

No datasets were generated or analysed during the current study.
