# Supplementary material for: Plasma levels of adhesion molecules are elevated in dermatomyositis-interstitial lung disease and associated with low paraoxonase-1 activity
Source: Arthritis Res Ther. 2025 Mar 8;27:53. doi: 10.1186/s13075-025-03520-z (PMC11889823; doi:10.1186/s13075-025-03520-z)
Supplement: Supplementary file 1 — Supplementary Material 1 [file 13075_2025_3520_MOESM1_ESM.docx]

**Supplementary Table 1. Multivariate Linear Regression Models of plasma CAM levels in DM and Controls (n=111)**

|  | **ICAM-1** | | **VCAM-1** | |
| --- | --- | --- | --- | --- |
|  | $\boldsymbol{\beta}$ **[95%CI]** | **P value** | $\boldsymbol{\beta}$ **[95%CI]** | **P value** |
| DM (vs control) | **105.15[8.31,202.01]** | **0.03** | **0.33 [0.07, 0.58]** | **0.01** |
| ESR per 10mm/hr | 10.92[-4.02,25.85] | 0.15 | -0.003[-0.04, 0.04] | 0.87 |
| Triglyceride per 100 | **24.18[5.20,43.16]** | **0.01** | **0.06[0.01, 0.11]** | **0.03** |
| Diabetes | 63.83[-97.76,225.43] | 0.43 | 0.20[-0.23,0.62] | 0.35 |

$\beta$ [95%CI] Regression coefficient and 95% confidence interval

ICAM-1 and VCAM-1 levels were log transformed to fit linearity. hsCRP was colinear with ESR

**Supplementary Table 2. MSA subgroups in DM and controls (n=111)**

|  | **ICAM-1** | | | | | | | **VCAM-1** | | | | | |
| --- | --- | --- | --- | --- | --- | --- | --- | --- | --- | --- | --- | --- | --- |
|  | $\boldsymbol{\beta}$ | **P value** | $\boldsymbol{\beta}$ | **P value** | $\boldsymbol{\beta}$ | **P value** | $\boldsymbol{\beta}$ | | **P value** | $\boldsymbol{\beta}$ | **P value** | $\boldsymbol{\beta}$ | **P value** |
| Anti-Jo1 DM | 0.15 | 0.08 | **-** |  | - | - | 0.18 | | 0.15 | - |  | - | - |
| Anti-MDA5 DM | - | - | **0.18** | **0.04** | - | - | - | | - | **0.29** | **0.02** | - | - |
| Anti-TIF1$\gamma$ DM | - | - | - | - | -0.11 | 0.17 | - | | - | **-** |  | -0.13 | 0.25 |
| ESR per 100mm/hr | **0.03** | **0.03** | 0.24 | 0.07 | **0.31** | **0.02** | 0.12 | | 0.53 | 0.03 | 0.87 | 0.01 | 0.46 |
| Triglyceride per 100 | **0.03** | **0.06** | **0.04** | **0.02** | **0.04** | **0.03** | **0.05** | | **0.04** | **0.06** | **0.01** | **0.05** | **0.02** |
| Diabetes | 0.12 | 0.41 | 0.11 | 0.47 | 0.11 | 0.46 | 0.19 | | 0.38 | 0.16 | 0.02 | 0.18 | 0.42 |

$\beta$ [95%CI] Regression coefficient and 95% confidence interval

Anti-Jo1 n=24, anti-MDA5 n=29, anti-TIF1r $\gamma$ n=30

ICAM-1 and VCAM-1 levels were log transformed to fit linearity. hsCRP was colinear with ESR

**Supplementary Table 3. Comparison in ILD vs no ILD DM (n=83)**

|  | **ILD**  **(n=42)** | **No ILD**  **(n=41)** | **P value** |
| --- | --- | --- | --- |
| Age | **52** $\boldsymbol{\pm}$**15*** | 43$\pm$115 | **0.01** |
| Sex, female | **28 (67)*** | 37 (90) | **0.01** |
| Ethnicity, Hispanic | 7(17) | 5(12) | 0.56 |
| Race, White | 25(60) | 33(80) | 0.10 |
| Black | 3(7) | 2(5) |  |
| Asian | 14(33) | 6(15) |  |
| ICAM-1, ng/mL | **576[491-764]*** | 394[336-509] | **<0.001** |
| VCAM-1, ng/mL | **3350[2730-4939]*** | 2557[1923-3687] | **0.01** |
| Paraoxonase, U/ml | **381[233-739]*** | 696[324-1063] | **0.04** |
| Arylesterase, U/ml | 199[152-264] | 206[160-294] | 0.43 |
| Lactonase, U/ml | 14[10-21] | 18[11-22] | 0.37 |
| hsCRP, mg/L | **2.6[0.9-9.4]*** | 0.9[0.4-4] | **0.01** |
| ESR, mm/hr | 30[14-52] | 23[9-42] | 0.22 |
| Total cholesterol, mg/dl | 213[178-251] | 204[175-241] | 0.31 |
| LDL-C, mg/dl | 117[93-169] | 118[89-150] | 0.82 |
| HDL-C, mg/dl | 54[41-73] | 62[53-80] | 0.06 |
| Triglyceride, mg/dl | **181[119-302]*** | 125[83-172] | **0.004** |
| *CVD risk factors* |  |  |  |
| History of MI | 1(2) | 0 | 0.24 |
| History of stroke | 2(5) | 2(5) | 0.98 |
| Hypertension | 8(19) | 8(19) | 0.96 |
| Hyperlipidemia | 4(10) | 6(14) | 0.47 |
| Diabetes | **8(19)*** | **0** | **0.001** |
| FHx of premature MI | 4(12) | 2(7) | 0.54 |
| Ever smoker | 7(19) | 5(17) | 0.82 |
| BMI, kg/m2 | 26[23-30] | 27[23-30] | 0.80 |
| Statin | 5(12) | 4(10) | 0.75 |
| ***IIM characteristics*** |  |  |  |
| Disease duration from symptom onset, months | 14[8-37] | 27[8-82] | 0.15 |
| MSA group, Jo-1 | 21(50) | 3(7) | **<0.01** |
| MDA5 | 21(50) | 8(20) | **<0.01** |
| TIF1r | 0 | 30(73) | **<0.01** |
| Cancer | 6(14) | 9(22) | 0.36 |
| MD activity VAS | 50[36-66] | 45[28-60] | 0.15 |
| MD activity likert | 2[2-3] | 2[1-2 | 0.08 |
| MD damage VAS | **45[32-54]*** | 13[6-30] | **<0.01** |
| MD damage likert | **2[1-2]*** | 1[1-1] | **<0.01** |
| MMT8 | 148[142-150] | 150[145-150] | 0.12 |
| CPK, U/ml | 68[45-182] | 79[61-124] | 0.56 |
| Aldolase, U/ml | 6.6[5.3-9.3]* | 5.6[3.9-7.4] | **0.03** |
| *Medications,* at visit |  |  |  |
| Intravenous or subcutaneous immunoglobulin | 21(50) | 16(39) | 0.31 |
| Mycophenolate Mofetil | **24[57]*** | 12[29] | **0.01** |
| Rituximab | **14(33)*** | 4(10) | **0.01** |
| Cyclophosphamide | **6(14)*** | 0 | **<0.01** |
| Azathioprine | 3(7) | 1(3) | 0.32 |
| Methotrexate | **1(2)*** | 13(32) | **<0.01** |
| Hydroxychloroquine | 5(12) | 11(27) | 0.08 |
| Prednisone | **36(86)*** | 26(63) | **0.02** |

Values are in mean $\pm$SD for non-skewed data, median[IQR] for skewed data

*p<0.05 by student’s t test for non-skewed data and Wilcoxon test for skewed data, chi square test for categorical data

**Supplementary Table 4. Association between CAM levels and ILD severity using Multivariate linear regression in DM only cohort**

|  | **FVC** | | | | **DLCO** | | | |
| --- | --- | --- | --- | --- | --- | --- | --- | --- |
|  | $\boldsymbol{\beta}$ | **P value** | $\boldsymbol{\beta}$ | **P value** | $\boldsymbol{\beta}$ | **P value** | $\boldsymbol{\beta}$ | **P value** |
| ICAM-1 | **-17.49** | **0.04** | - | - | **-18.3** | **0.01** | - | - |
| VCAM-1 | - | - | 1.04 | 0.88 | - | - | -0.56 | 0.93 |
| Age | -0.24 | 0.26 | -0.22 | 0.32 | -0.33 | 0.07 | -0.31 | 0.11 |
| Sex, Female | 7.53 | 0.32 | 8.66 | 0.29 | -4.46 | 0.50 | -3.09 | 0.67 |
| Diabetes | -6.56 | 0.58 | -10.21 | 0.42 | 4.22 | 0.71 | -0.97 | 0.93 |
| Triglyceride per 100 | -0.26 | 0.84 | -0.95 | 0.48 | -0.19 | 0.87 | -0.80 | 0.49 |
| MMF | 2.90 | 0.65 | 3.41 | 0.62 | -6.36 | 0.27 | -5.00 | 0.42 |

$\boldsymbol{\beta}$ Regression coefficient, ICAM-1 and VCAM-1 were log transformed

Includes patients with PFT values available, 35 patients with ILD and 24 without ILD

Model including MMF reported. Models including other medications show similar significance for CAM

**Supplementary Table 5. Association between Lactonase and CAM levels using Multivariate linear regression in DM only (n=83)**

|  | **Log ICAM** | | **Log VCAM** | |
| --- | --- | --- | --- | --- |
|  | $\boldsymbol{\beta}$ **[95%CI]** | **P value** | $\boldsymbol{\beta}$ **[95%CI]** | **P value** |
| **Lactonase**, 10U/L | **-0.15[-0.28,-0.03]** | **0.02** | -0.01[-0.17,0.15] | 0.91 |
| Disease duration, year | -0.004[-0.02,0.02] | 0.69 | - | - |
| Aldolase, 10U/L | **0.04[0.01,0.07]** | **0.02** | - | - |
| MD damage VAS,10mm | 0.01[-0.05,0.07] | 0.68 | - | - |
| FVC,10%predicted | -0.03[-0.08,0.03] | 0.31 | - | - |
| Methotrexate | **-0.46[-0.79,-0.13]** | **0.01** | -0.29[-0.64,0.07] | 0.11 |
| Prednisone dose | - | - | 0.004[-0.003,0.01] | 0.27 |
| Dyspnea | - | - | 0.11[-0.21,0.42] | 0.45 |
| Fever | - | - | 0.01[-0.38,0.39] | 0.97 |

Multivariate models adjusted for variables significantly associated with CAM (Table 3). When variables were colinear, one representative variable was selected. Continuous variables were preferentially selected (ex, FVC over ILD yes/no)

**Supplementary Figure 1. Schema for causal mediation analysis.**


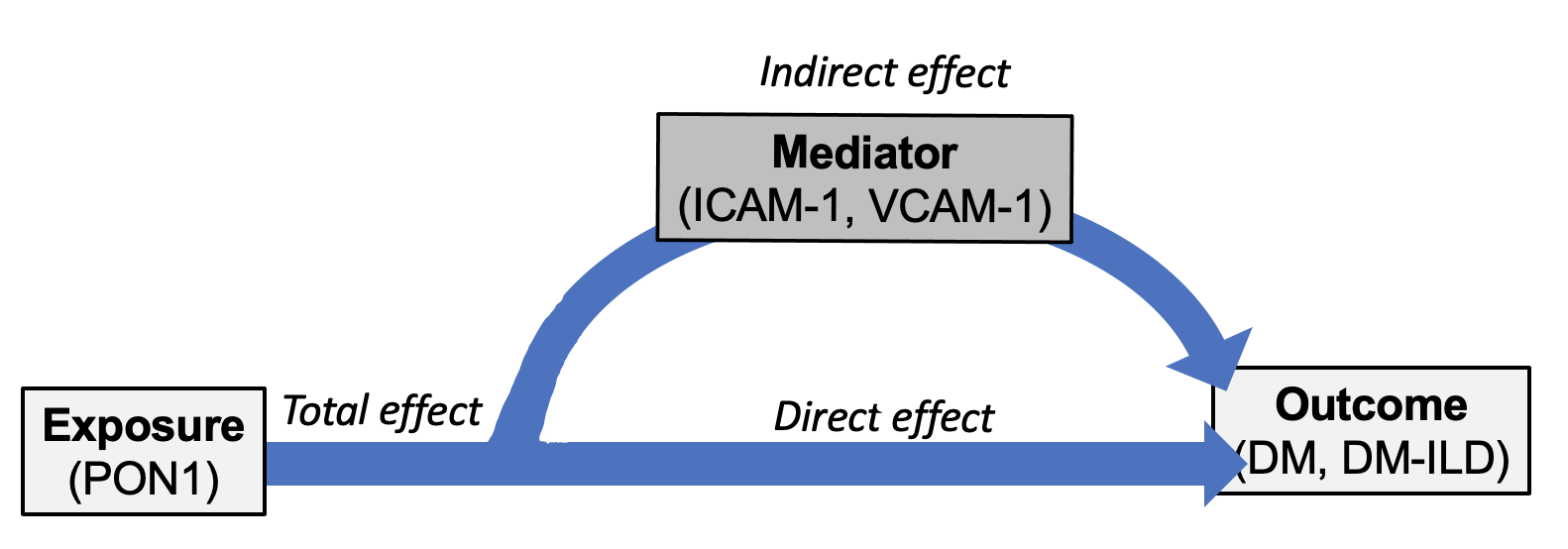


**Supplementary Figure 2. Correlation between PON1 activity by lactonase and CAM levels in DM patients and controls (n=111)**

| 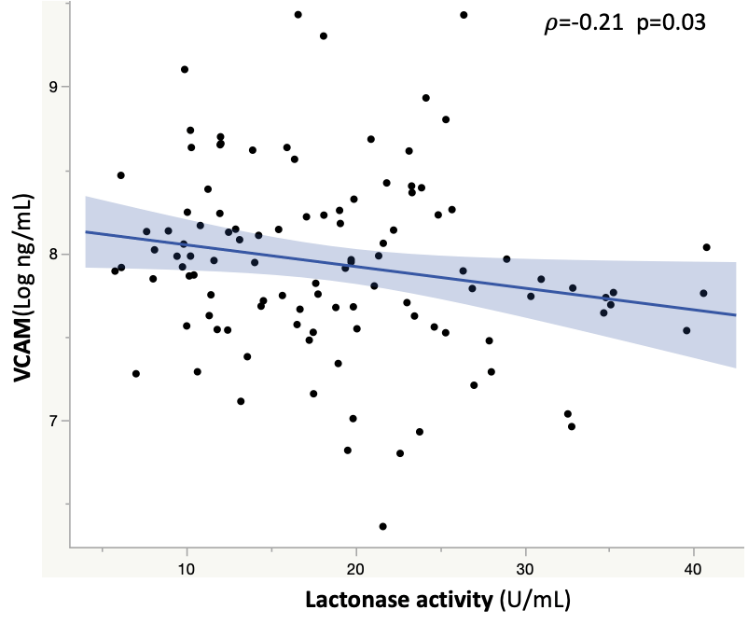 | 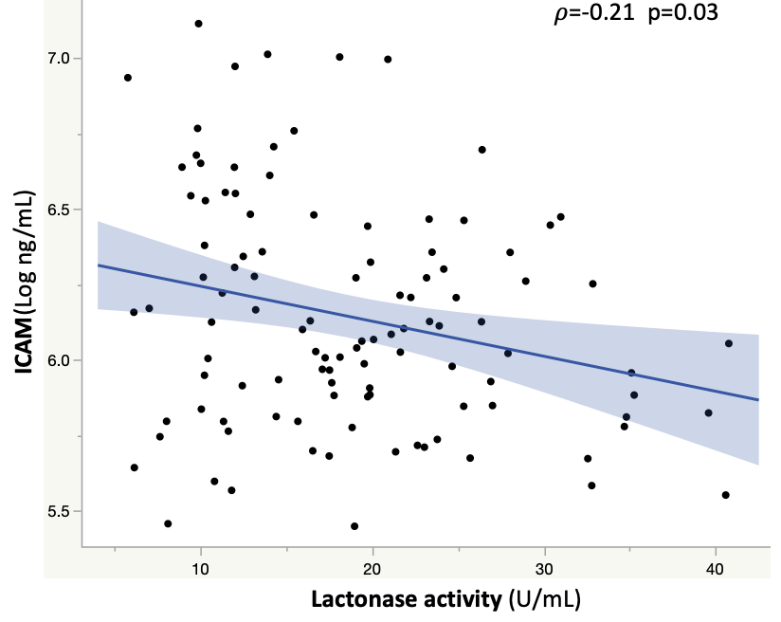 |
| --- | --- |
| 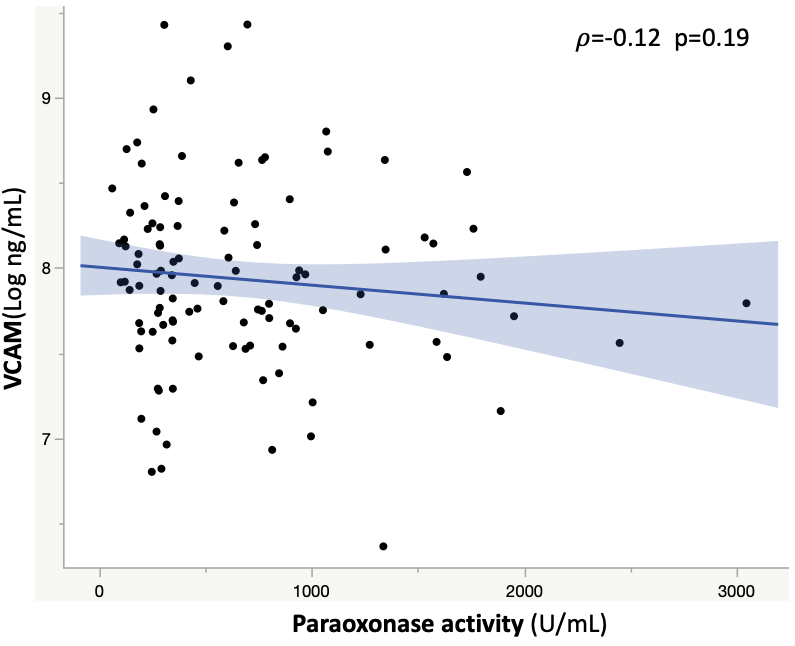 | 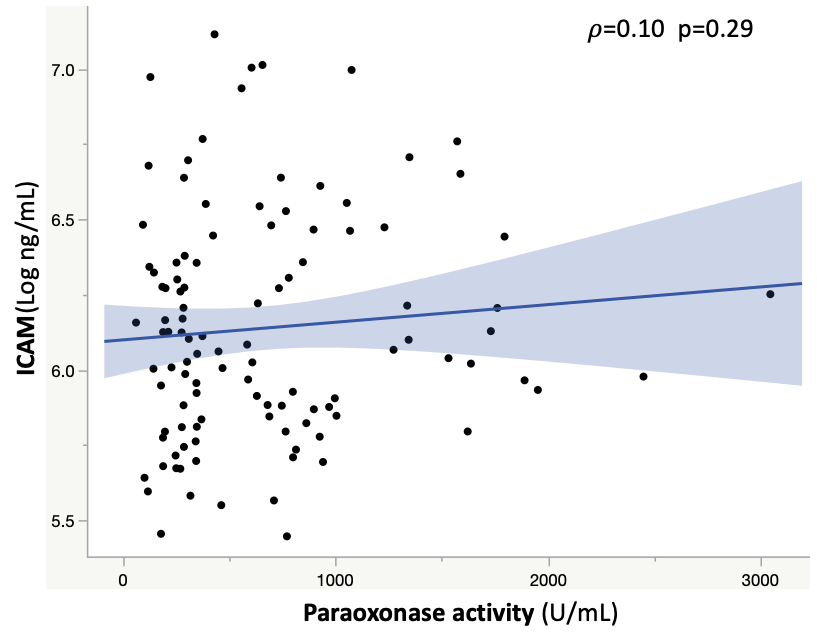 |
| 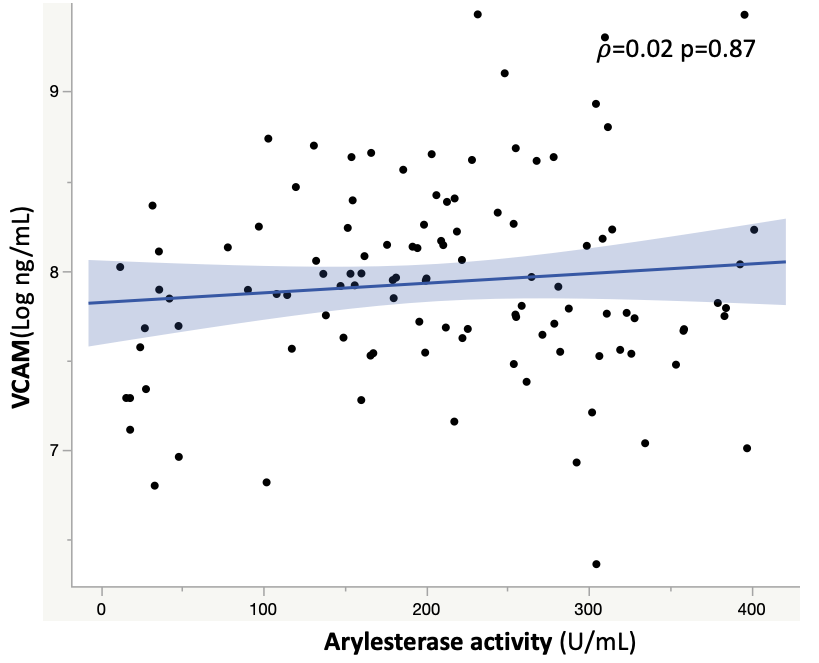 | 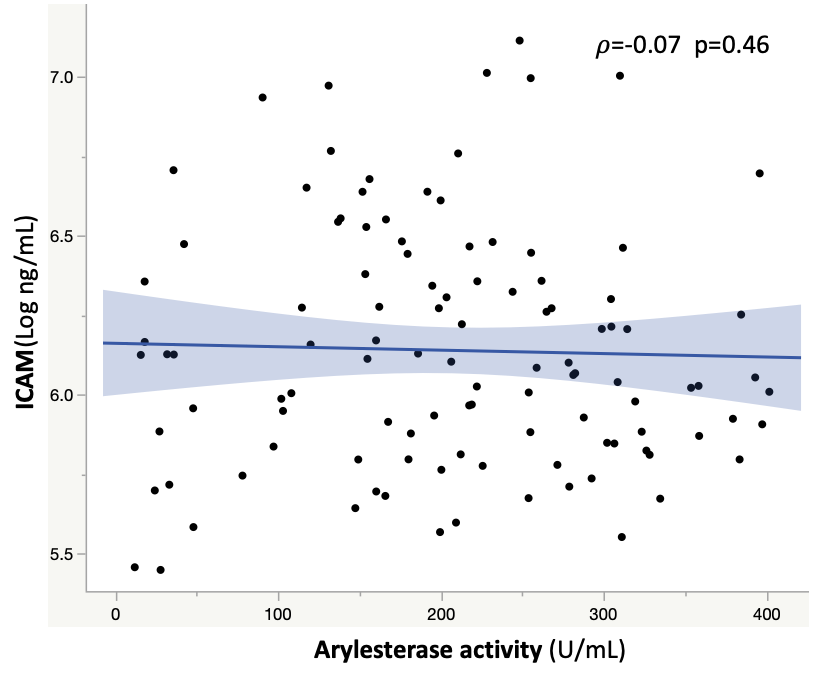 |

Spearman correlation coefficient ($\rho$) between PON1 by lactonase with log transformed CAM levels
